# Introducing the J-CONNECT database: a real-world oncology resource for Japan

**DOI:** 10.1007/s10147-026-03011-4

**Published:** 2026-04-24

**Authors:** Masafumi Okada, Shigemi Matsumoto, Yuriko Maehara, Tomoko Kanayama, Dimitra Lambrelli, Tadashi Koga, Manabu Muto

**Affiliations:** 1 Prime Research Institute for Medical RWD, Inc., Kyoto, Japan; 2 Department of Real World Data R&D, Graduate School of Medicine, Kyoto University, Kyoto, Japan; 3 PPD Evidera Real-World Data & Scientific Solutions, Thermo Fisher Scientific, London, UK; 4 Clinical Study Support, Inc., Nagoya, Japan; 5 Department of Medical Oncology, Graduate School of Medicine, Kyoto University, Kyoto, Japan

**Keywords:** Real-world data, Electronic medical records, Oncology, Japan

## Abstract

**Background:**

Real-world data (RWD) and real-world evidence (RWE) are increasingly important in oncology, where randomized controlled trials often exclude elderly patients, those with comorbidities, and rare cancer subtypes. To meet the need for high-quality oncology RWD in Japan, the J-CONNECT Consortium was launched to build a multiinstitutional database of chemotherapy-treated solid cancer patients using electronic medical records (EMRs).

**Methods:**

Data were collected from 12 Ministry of Health, Labour and Welfare–designated cancer care hospitals, with expansion to 15 institutions planned by 2026. J-CONNECT links hospital cancer registry information with EMRs, capturing demographics, staging, treatments, laboratory results, and biomarkers. Coverage and distributional characteristics were evaluated by comparing distributions with the National Hospital-based Cancer Registry by cancer site, age, and sex.

**Results:**

The database included 51,497 patients diagnosed after 2018. Major cancer groups were breast (12,363), lung (6743), prostate (5235), colorectal (4012), pancreas (3720), stomach (2383), esophagus (1960), and liver (1731). Sex distributions were consistent with national data, while some variability was observed by age.

**Conclusion:**

J-CONNECT is a robust oncology database in which clinical and biomarker variables are available, although completeness may vary; fitness-for-purpose should be assessed on a study-by-study basis. Coverage and distributional characteristics and also regulatory acceptance highlight its value for research, PMS, and policy. Future directions include expansion to 25 hospitals, integration of outcome and resource-use data, and adoption of common data models for broader collaboration. We believe J-CONNECT provides valuable oncology data supporting research, post-marketing surveillance, and public health policy in Japan.

**Supplementary Information:**

The online version contains supplementary material available at 10.1007/s10147-026-03011-4.

## Introduction

In recent years, the role of real-world data (RWD) and real-world evidence (RWE) in the field of oncology has been expanding to guide decision-making at both clinical and policy levels. Although randomized controlled trials (RCTs) are considered the gold standard for evaluating the efficacy of medical interventions, they are conducted under highly controlled conditions with carefully selected patient populations, resulting in a gap from real-world clinical practice [1, 2] and limited external validity. The external validity of RCTs is particularly limited for elderly patients, those with comorbidities and patients with rare molecular subtypes of cancer. In contrast, RWE derived from RWD sources, including electronic medical records (EMRs), cancer registries, and claims data, reflects the diversity of patient clinical profiles, backgrounds, and treatment patterns in routine clinical practice. Applying appropriate analytical methods to RWD makes it possible to clarify aspects such as real-world treatment practices, effectiveness, long-term outcomes, and safety in real-world settings, which are factors that are difficult to fully evaluate through RCTs [3, 4].

RWE is also increasingly being utilized to complement regulatory decision-making, such as serving as external controls in single-arm trials for rare diseases including rare cancers [5]. Methodological recommendations from multistakeholder initiatives further support oncology use cases [6]. In Japan, the Pharmaceuticals and Medical Devices Agency (PMDA) now accepts secondary RWD-based post-marketing surveillance (PMS) studies as part of regulatory submissions [7, 8]. This acceptance has been facilitated by the issuance of national guidelines (e.g., the Ministry of Health, Labour and Welfare (MHLW)’s “Basic Principles on Utilization of Registry for Applications” and “Basic principles on the use of medical information databases in post-marketing pharmacovigilance”) [9, 10].

### Background to the development of J-CONNECT

In Japan, cancer care is predominantly delivered in hospital settings, including designated cancer care hospitals accredited by MHLW [11]. These institutions are responsible for providing standardized cancer treatment and are equipped with multidisciplinary teams, including oncologists, surgeons, radiologists, and palliative care specialists.

The J-CONNECT Consortium was established in April 2023 in consideration of the above care environment, with the aim of generating high-quality RWE in the field of oncology in Japan.

J-CONNECT is a collaborative academic network in Japan that has developed a real-world database of chemotherapy-treated solid cancer patients using EMRs. J-CONNECT uses CyberOncology, an EMR template optimized for cancer research data collection. It utilizes automatic data extraction from EMR systems which significantly streamlines the data collection process. Of note, CyberOncology has been approved by the PMDA for the generation and submission of PMS data supporting the long-term safety of medicines and devices [12]. It is envisioned that this approval will facilitate the use of J-CONNECT data in providing long-term PMS data inexpensively and efficiently.

Additionally, J-CONNECT serves as a multiinstitutional research platform, incorporating participation from numerous bodies including pharmaceutical companies.

Overall, therefore, the J-CONNECT database was developed to provide access to selected clinical variables (e.g., treatment details, laboratory tests, and biomarkers) captured in routine care settings at participating hospitals, while recognizing that completeness may vary across variables and settings.

### Unique features and research enablement of J-CONNECT

J-CONNECT is designed to complement existing Japanese cancer registries and administrative datasets by providing higher clinical granularity within participating hospitals. Key differentiators include: (1) regimen- and line-of-therapy-level treatment information for chemotherapy-treated patients, (2) structured capture of selected biomarkers and laboratory test results to support biomarker-driven analyses and safety monitoring, and (3) a multicenter framework with a common data dictionary and governance to facilitate consistent cross-site data harmonization. These features enable research questions that are difficult to address using claims data alone, including detailed treatment sequencing and switching patterns, biomarker-guided treatment selection and uptake, and laboratory-based safety surveillance in routine clinical practice; however, completeness is variable and should be evaluated for each study purpose.

### Objectives

The objective of this article is to introduce the J-CONNECT database, to describe its strengths in generating RWE oncology data; demonstrate its coverage and distributional characteristics by comparing the data with National Hospital-based Cancer Registry data and to present future perspectives that contribute to the advancement of RWD utilization in the field of oncology.

## Patients and methods

### Source and coverage

#### Data sources and participating institutions

Data from twelve MHLW designated cancer care hospitals are currently available for analysis. The network of contributing cancer care hospitals is continuing to expand and it is planned to include fifteen cancer care hospitals in the J-CONNECT Consortium by the end of March 2026. Institutions include both university-affiliated and nonuniversity hospitals, under national, prefectural, municipal, and private governance. Geographic coverage spans from Hokkaido in the north of Japan to Kyushu in the south, reflecting nationwide representation in Japan.

#### Data sources and patients

The National Hospital-based Cancer Registry database is a system in Japan for the standardized recording and management of information on cancer patients diagnosed and treated at medical institutions. All cancer patients are included, and approximately 100 data elements are registered, such as cancer type, stage, and treatment details. The system is mainly operated by MHLW designated cancer care hospitals, which report data annually to the National Cancer Center (NCC). Although not legally mandated, registration is effectively required for these designated hospitals.

Records are created in accordance with the rules of the National Hospital-based Cancer Registry. Data are entered, at the time of diagnosis and treatment, based on a nationally standardized registration format, using dedicated software. Approximately 100 data items are accurately coded using international classification standards such as International Classification of Diseases for Oncology, Third Edition (ICD-O-3) and Tumor, Node and Metastasis (TNM) staging. The data are anonymized and submitted to the NCC annually. Regular training and quality control measures are conducted to ensure data accuracy.

One record is created for each cancer type; therefore, patients with multiple primary cancers will have multiple records for each cancer type. Data on recurrences are continuously added to the same record for each patient.

The J-CONNECT cohort for this database profile is defined as follows: first, we identify patients with solid tumors registered in the National Hospital-based Cancer Registry at participating hospitals. From this registry-based solid tumor population, we include patients who received at least one administration of any systemic anti-cancer drug during the observation period at the participating hospital (“chemotherapy-treated”). Systemic anti-cancer drugs are identified from medication administration/ordering records in the EMR-derived dataset and are mapped to a harmonized drug list according to the common data dictionary.

#### Collection period, data update frequency

Patients newly diagnosed with solid cancer are registered once a year.

Chronological data including drug data, laboratory test records, and biomarker test records are available from January 1, 2018, to the present day. Medical data for all patients who have been registered and included in the database are updated real-time on a daily basis.

#### Data extraction, harmonization, and quality control

**Fig. 1:**
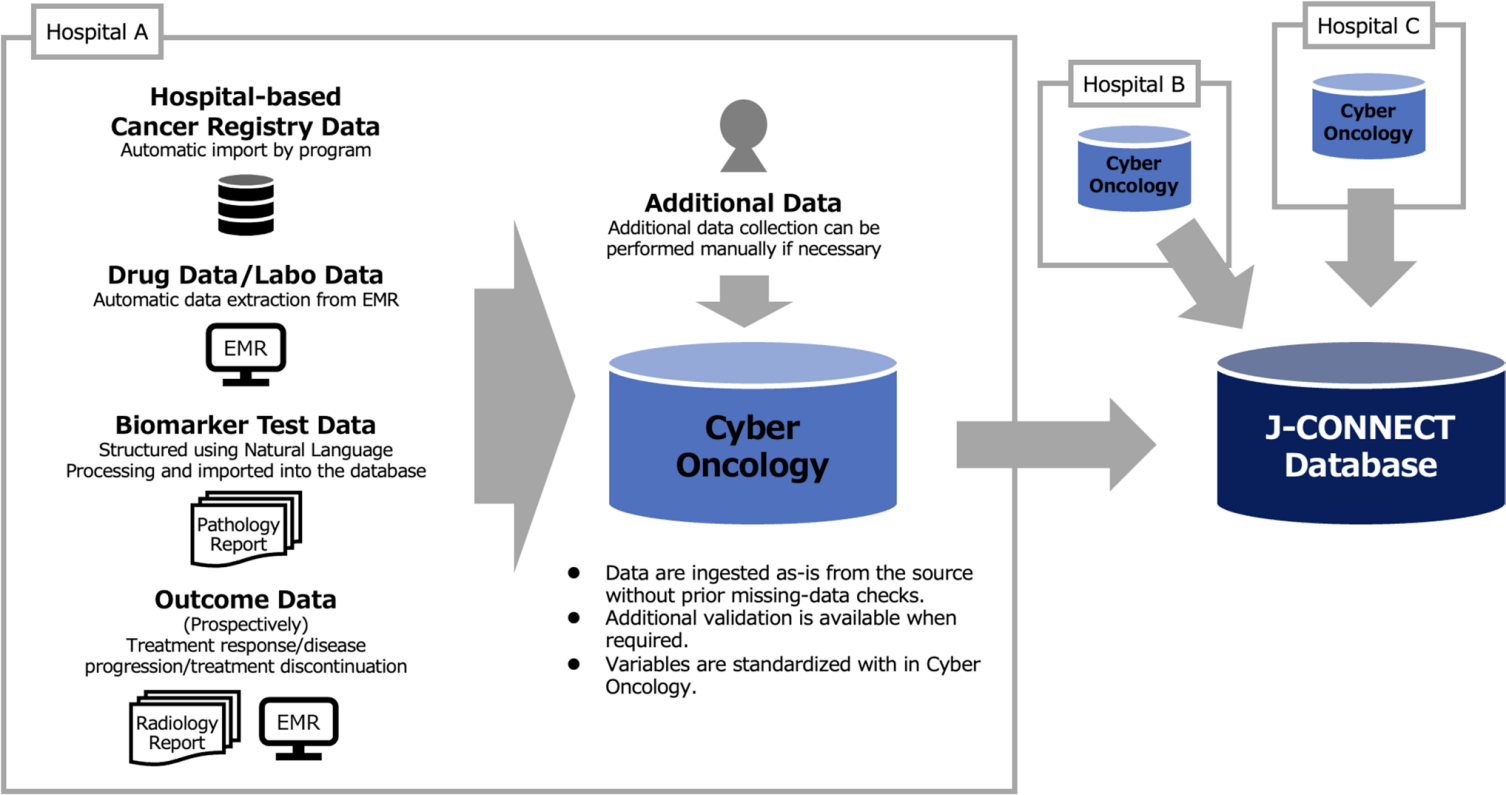
Data extraction, harmonization, and quality control workflow for J-CONNECT

J-CONNECT is derived from routinely collected EMR data and hospital-based cancer registry (HBCR) data at participating hospitals (Fig. 1). Within each hospital, EMR domains (e.g., drug administration, laboratories) and HBCR records are linked deterministically using the internal patient identifier in the CyberOncology platform, and transferred to the central J-CONNECT database under appropriate governance. Variables are harmonized using a consortium-maintained common data dictionary, including standardization of variable definitions, coding conventions, and (where feasible) normalization of laboratory identifiers/units and anti-cancer drug mappings. Laboratory tests and biomarkers are available only when requested/measured and recorded in routine care; otherwise, fields are missing. Missingness is preserved (no imputation in this database profile) and QC includes completeness checks for required fields and plausibility/consistency checks (e.g., ranges/units, duplicates, and temporal sequencing).

### Database structure and encryption of personal identifiers

In J-CONNECT for cancer-type data, one record is created per patient. For treatment and laboratory test data, multiple records per patient are maintained. Each data element is managed in a table-based structure. Each patient has a unique pseudonymized ID enabling linkage of different files, including cancer diagnoses files, treatment files, and laboratory test files. The correspondence table linking personal identifiers to pseudonymized IDs is securely stored within each medical institution.

### Data components

The database includes core cancer registration data, as well as detailed treatment data, and laboratory test records extracted from EMRs. A nonexhaustive list of data elements that are collected can be found in Table 1, and curated structured clinical laboratory evaluation parameters are listed in Table 2. For more detailed analyses, researchers can access additional data, including adverse events and complications, through a federated model or by directly collaborating with individual hospitals.

**Table 1:** Data collection items

Category	Data source	Data item	Remarks
Patient information	Hospital-based Cancer Registry	Age	Calculated from date of birth
	Hospital-based Cancer Registry	Sex	
Disease information	Hospital-based Cancer Registry	Date of Diagnosis	
	Hospital-based Cancer Registry	Cancer type (detailed location/pathological tissue)	ICD-O-3
	Hospital-based Cancer Registry	Clinical stage	UICC TNM classification
Treatment information	Hospital-based Cancer Registry	Date of initial chemotherapy	
	Electronic Medical Record	Drug data (prescription date/drug code/dosage/unit/duration)	YJ code/ATC code
	Electronic Medical Record	Laboratory test results (test date/test code/result)	CTCAE version 5.0
	Pathology Report	Biomarker test data (biomarker name/specimen collection or receipt date/testing method/result)	
Outcome	Hospital-based Cancer Registry	Survival and death information (date of death/last confirmation of survival)	In-hospital death only

**Table 2:** clinical laboratory evaluation parameters

Abbreviation	Laboratory test item
HGB	Hemoglobin
WBC	White blood cell count
PLT	Platelet count
ALT/GPT	Alanine aminotransferase (ALT/GPT)
UA	Uric acid
AST/GOT	Aspartate aminotransferase (AST/GOT)
ALP	Alkaline phosphatase (ALP)
T-Bil	Total bilirubin (TB)
K	Potassium
ALB	Albumin
CRE	Creatinine
γ-GTP	Gamma-glutamyl transpeptidase (γ-GT)
T-cho	Total cholesterol (TC)
Na	Sodium
LYMPH#	Absolute lymphocyte count
NEUT#	Absolute neutrophil count
Ca	Calcium
APTT	Activated partial thromboplastin time (APTT)
CK	Creatine kinase (CK)
FIB	Fibrinogen (Fbg)
Haptoglobin	Haptoglobin (Hp)
PT(INR)	Prothrombin time INR
Lipase	Lipase
AMY	Amylase
pH	Blood gas pH (hydrogen ion concentration)
Glucose	Blood glucose
Mg	Magnesium (Mg)
IP	Inorganic phosphorus (IP)
TG	Triglycerides (TG)
Protein (qual.)	Urine protein (qualitative)
Eosinophils	Eosinophil count
HCO3-act	Bicarbonate ion (HCO₃⁻ act) in blood gas
LDH	Lactate dehydrogenase (LD)
eGFR	Estimated glomerular filtration rate (eGFR)

### Governance access and applications

This database was constructed by the J-CONNECT Consortium, led by Kyoto University Hospital and PRiME-R (Prime Research Institute for Medical RWD, Inc.). The database is constructed under an academic framework with an opt-out informed consent process. It complies with Japanese laws and regulations regarding the protection of personal information.

### Analysis methods

Analysis was conducted to assess the comparability of J-CONNECT data with the National Hospital-based Cancer Registry. In this analysis, the coverage of database cases was calculated by cancer site. Stratified coverage by age and gender was evaluated for stomach, esophagus, colon, lung, prostate, breast, liver, biliary tract (including gallbladder), and pancreas cancers using publicly available national cancer registry data. The standardized difference (SMD) was used to assess distributional comparability across strata.

## Results

Table 3 shows the comparison of the number of cancer cases identified by cancer site in J-CONNECT, compared to the number of cases in the National Hospital-based Cancer Registry database. The Coverage Rate of J-CONNECT sites is the percentage of cases compared to the National Hospital-based Cancer Registry database (number of cases in J-CONNECT). The J-CONNECT Coverage rates in descending order were as follows: liver 3.42% (1731), esophagus 3.33% (1960), breast 3.30% (12,363), pancreas 3.28% (3720), lung 2.91% (6743), prostate 2.26% (5235), stomach 2.21% (2383), and colorectal 2.00% (4012).

**Table 3:** Comparison of J-CONNECT case populations by cancer site compared to the National Hospital-based Cancer Registry (2018–2023)

Cancer site	J-CONNECT count	National count	J-CONNECT coverage rate
Liver	1731	50,547	0.0342
Esophagus	1960	58,804	0.0333
Breast	12,363	374,907	0.0330
Pancreas	3720	113,436	0.0328
Lung	6743	231,478	0.0291
Prostate	5235	232,128	0.0226
Stomach	2383	107,623	0.0221
Colorectal	4012	200,730	0.0200

Only cancer sites comparable to the National Hospital-based Cancer Registry for coverage rate were included. The data collection period was 2018–2023

**Fig. 2:**
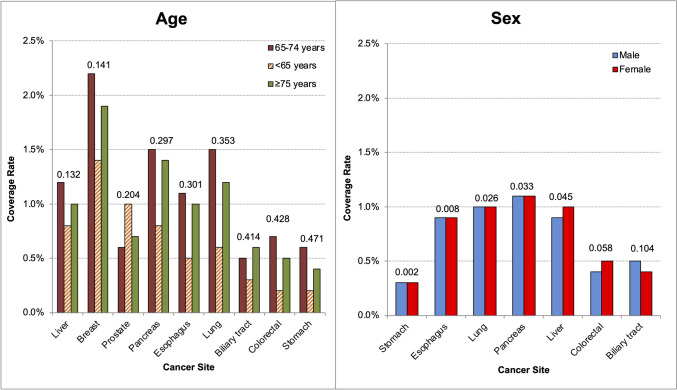
J-CONNECT coverage rate by cancer site and subgroup type. The maximum absolute standardized difference (SMD) across strata is shown for each cancer site; smaller absolute SMD indicates less deviation from national tabulations

Figure 2 and Supplemental Table S1 show the comparison of the number of age-stratified (< 65 years; 65–74 years; ≥ 75 years) cancer cases identified by cancer site in J-CONNECT, compared to the number of age-stratified cases identified by cancer site in the National Hospital-based Cancer Registry database. The maximum absolute SMD of each age strata in ascending order was as follows: liver (0.132), breast (0.141), prostate (0.204), pancreas (0.297), esophagus (0.301), lung (0.353), biliary tract (0.414), colorectal (0.428), and stomach (0.471). These larger absolute SMDs indicate greater distributional differences between J-CONNECT and the national tabulations when stratified by age.

Figure 2 and Supplemental Table S2 compare the numbers of cancer cases identified at J-CONNECT participating sites and stratified by cancer site and sex with those in the National Hospital-based Cancer Registry database. The absolute SMDs in ascending order (excluding breast and prostate) were as follows: stomach (0.002), esophagus (0.008), lung (0.026), pancreas (0.033), liver (0.045), colorectal (0.058), and biliary tract (0.104). These results suggest that the sex-specific distribution of cancer sites in J-CONNECT is closely aligned with the corresponding national tabulations, whereas age-stratified distributions show larger differences.

## Discussion

The J-CONNECT Consortium was established in April 2023. This article introduces J-CONNECT and summarizes its coverage and distributional characteristics against publicly available national tabulations, with future perspectives for RWE in oncology.

### Strengths of J-CONNECT

#### Broad coverage and standardized data capture

The database incorporates hospital-based cancer registry records for cases diagnosed within participating institutions. The key variables such as date of diagnosis, TNM staging, and histological subtype are entered by trained specialists and submitted through a standardized registry system, which is expected to support consistent data capture and quality [13].

#### Biomarker variables captured in routine care

Important oncology biomarkers (e.g., EGFR and ALK) are captured when tested in routine clinical practice, although completeness varies by cancer type, time period, and institution.

#### Integration with electronic medical records (EMRs)

EMR integration enables capture of regimen-based treatment and administration information and selected laboratory parameters (e.g., ALT, AST, and creatinine) where recorded. Completeness may vary; fitness for purpose should be assessed for each study question. For reference, to assess feasibility, the data availability of selected laboratory variables is quantified (Supplementary Table S3). Availability is defined as the presence of at least one recorded value within 30 days of the initial diagnosis date.

#### Possibility of additional data collection

For patients registered in the database, additional data can be collected, as required, using correspondence tables stored securely at each institution. This feature enables the use of this data in more detailed clinical research and post-marketing surveillance (PMS) studies.

#### Multicenter and nationwide coverage

Data are collected from twelve institutions, including university hospitals and cancer centers across Japan, ensuring geographic, institutional, and patient-level diversity.

#### Coverage and distributional characteristics versus national tabulations

To contextualize the captured population, J-CONNECT can be benchmarked against published National Hospital-based Cancer Registry tabulations using transparent distributional diagnostics (Fig. 2; Tables S1–S2). In our comparisons, sex-stratified distributions were generally closer to national tabulations (absolute SMD range 0.002–0.104), whereas age-stratified distributions showed larger deviations (maximum absolute SMD range 0.132–0.471). These patterns are consistent with J-CONNECT’s treatment-exposed cohort definition and participating site case mix; accordingly, the comparisons are intended as descriptive diagnostics rather than evidence of national representativeness.

#### Scientific implications and research use cases

J-CONNECT can support analyses that leverage clinical variables captured in routine practice, particularly for questions centered on systemic anti-cancer treatment patterns. However, completeness of EMR-derived variables (including laboratory tests and biomarkers) may vary and should be assessed on a study-by-study basis. First, it supports treatment pathway characterization at regimen and line-of-therapy levels (e.g., sequencing, switching, and combination patterns) across multiple institutions. Second, the availability of selected biomarkers and laboratory test results allows evaluation of biomarker-driven treatment uptake and laboratory-informed safety characterization in real-world settings. Third, J-CONNECT can support pharmacovigilance and safety characterization using EMR-derived measurements (e.g., laboratory abnormalities) and treatment exposures, where endpoint definitions align with data captured within participating hospitals. Finally, the database can be used for feasibility assessments for external control studies (e.g., evaluating whether key eligibility criteria and baseline covariates are sufficiently captured), while recognizing that endpoint completeness may require additional sources depending on the study question.

#### Policy implications and governance for RWE

As real-world evidence is increasingly considered in clinical and regulatory decision-making, transparent documentation of data provenance, harmonization, and governance is essential to support fitness-for-purpose assessments. J-CONNECT is designed with a multiinstitutional governance framework and a common data dictionary to promote consistent data interpretation across sites. The database profile presented here is intended to facilitate appropriate use by clarifying what is captured reliably, what is partially captured, and what is outside the current scope.

### Limitations of J–CONNECT

First, the database includes only clinical data obtained within the participating institutions. Information on treatment or care provided at other hospitals is generally not traceable.

Second, deaths occurring within the hospital are recorded, but deaths at home or in other facilities may not be captured; therefore, J-CONNECT alone is not sufficient for complete overall survival analyses without additional linkage.

Third, clinical outcomes such as recurrence, response, or progression are not directly coded in the database and must be defined through specific algorithms tailored to each study. Recurrence events and standardized tumor response assessments (e.g., RECIST-based response) are not systematically available across sites. Consequently, endpoints that require complete longitudinal follow-up, such as overall survival without external linkage, recurrence-free survival, or progression-free survival may be biased by incomplete follow-up and should be interpreted cautiously.

Fourth, since the data are primarily collected from university hospitals and cancer centers, generalizability to community clinics or general hospitals may be limited.

Fifth, J-CONNECT is EMR-derived; completeness of laboratory and biomarker variables is not uniform across patients or institutions and may vary with clinical indication and local practice; therefore, missing values should be interpreted primarily as “not measured/not recorded” rather than absence of a clinical feature.

### Future directions

This expansion is expected to increase sample size and broaden coverage within the participating hospital network, while we recognize that representativeness to nonparticipating community settings may remain limited depending on participating site types. In parallel, J-CONNECT aims to enhance the availability of clinically relevant variables by expanding the biomarker dataset and improving harmonization across institutions via the common data dictionary and quality control framework.

To strengthen fitness-for-purpose for effectiveness-oriented research, we plan to incorporate additional outcome-related data elements, including treatment response, disease progression, and reasons for treatment discontinuation, where feasible and consistently definable across sites. A further practical next step is to explore integration and/or linkage with complementary data sources, such as Diagnosis Procedure Combination (DPC) data and other administrative claims, and potentially vital statistics where feasible to improve longitudinal follow-up, outcome ascertainment beyond the participating hospitals, and characterization of healthcare utilization and resource allocation.

International context. International oncology RWD initiatives span EHR-aggregation platforms, curated oncology EHR datasets with endpoint development, and clinico-genomic consortia (e.g., CancerLinQ [14]; Flatiron [15]; GENIE [16]). In this context, J-CONNECT plans to adopt the OMOP Common Data Model to support multiinstitutional and international collaborative research, while recognizing current limitations for outcomes requiring follow-up beyond participating hospitals.

## Conclusion

Although J-CONNECT includes only patients who received systemic anti-cancer therapy, sex-stratified distributions by cancer site were closely aligned with published national tabulations. In contrast, larger differences were observed for age-stratified distributions, underscoring the influence of cohort definition and participating-site case mix. Accordingly, the national comparisons presented in this manuscript should be interpreted as descriptive diagnostics rather than evidence of national representativeness.

As J-CONNECT expands to include up to 25 MHLW-designated cancer care hospitals, the database will increase in sample size and coverage within the participating hospital network. With transparent documentation of data provenance and governance, J-CONNECT can support a range of real-world oncology research applications and inform policy discussions, while study questions and endpoints should be selected in accordance with the data captured within participating hospitals.

## Supplementary Information

Below is the link to the electronic supplementary material.

Supplementary file (DOCX 40 KB)

## Data Availability

The datasets generated during and/or analyzed in this paper are available from the corresponding author on reasonable request.
