# Supplementary material for: Introducing the J-CONNECT database: a real-world oncology resource for Japan
Source: Int J Clin Oncol. 2026 Apr 24;31(6):931–9. doi: 10.1007/s10147-026-03011-4 (PMC13201281; doi:10.1007/s10147-026-03011-4)
Supplement: Supplementary file 1 — Supplementary file (DOCX 40 KB) [file 10147_2026_3011_MOESM1_ESM.docx]

Supplementary Tables

Title: Introducing the J-CONNECT Database: A Real-World Oncology Resource for Japan

Journal: International Journal of Clinical Oncology

DOI: 10.1007/s10147-026-03011-4

Manuscript ID: IJCO-D-25-01377R3

Authors: Masafumi Okada, Shigemi Matsumoto, Yuriko Maehara, Tomoko Kanayama, Dimitra Lambrelli, Tadashi Koga, and Manabu Muto

Supplemental Table S1 Comparison of case distributions by cancer site and age group (2018 – 2021)

| **Cancer Site** | **Age Group** | **National Count** | **J-CONNECT Count** | **J-CONNECT Coverage Rate** | **Standardized Difference** |
| --- | --- | --- | --- | --- | --- |
| Liver | <65 years | 21,930 | 274 | 0.012 | 0.132 |
|  | 65-74 years | 41,385 | 408 | 0.010 | 0.019 |
|  | ≥75 years | 61,896 | 519 | 0.008 | -0.125 |
| Breast | <65 years | 240,654 | 5,278 | 0.022 | 0.127 |
|  | 65-74 years | 104,673 | 1,987 | 0.019 | -0.021 |
|  | ≥75 years | 87,504 | 1,267 | 0.014 | -0.141 |
| Prostate | <65 years | 45,539 | 273 | 0.006 | -0.121 |
|  | 65-74 years | 144,518 | 1,037 | 0.007 | -0.128 |
|  | ≥75 years | 146,208 | 1,515 | 0.010 | 0.204 |
| Pancreas | <65 years | 33,266 | 485 | 0.015 | 0.156 |
|  | 65-74 years | 53,538 | 726 | 0.014 | 0.159 |
|  | ≥75 years | 72,962 | 553 | 0.008 | -0.297 |
| Esophagus | <65 years | 28,665 | 322 | 0.011 | 0.156 |
|  | 65-74 years | 47,725 | 486 | 0.010 | 0.136 |
|  | ≥75 years | 44,667 | 245 | 0.005 | -0.301 |
| Lung | <65 years | 84,173 | 1,293 | 0.015 | 0.221 |
|  | 65-74 years | 180,774 | 2,195 | 0.012 | 0.155 |
|  | ≥75 years | 215,124 | 1,363 | 0.006 | -0.353 |
| Biliary tract | <65 years | 9,265 | 46 | 0.005 | 0.093 |
|  | 65-74 years | 21,680 | 136 | 0.006 | 0.359 |
|  | ≥75 years | 44,892 | 116 | 0.003 | -0.414 |
| Colorectal | <65 years | 164,003 | 1,127 | 0.007 | 0.285 |
|  | 65-74 years | 207,764 | 1,120 | 0.005 | 0.124 |
|  | ≥75 years | 247,232 | 587 | 0.002 | -0.428 |
| Stomach | <65 years | 68,808 | 415 | 0.006 | 0.307 |
|  | 65-74 years | 136,800 | 600 | 0.004 | 0.192 |
|  | ≥75 years | 194,773 | 365 | 0.002 | -0.471 |

*Only cancer sites comparable to the National Hospital-based Cancer Registry for coverage rate were included. Age-stratified national tabulations were publicly available for 2018–2021, whereas overall national counts used for Table 3 were available for 2018–2023; therefore, analytic windows differ across tables.

Supplemental Table S2 Comparison of case distributions by cancer site and sex (2018 – 2021)

| **Cancer Site** | **Sex** | **National Count** | **J-CONNECT Count** | **J-CONNECT Coverage Rate** | **Standardized Difference** |
| --- | --- | --- | --- | --- | --- |
| Stomach | Male | 279,072 | 965 | 0.003 | -0.002 |
|  | Female | 121,309 | 421 | 0.003 | 0.002 |
| Esophagus | Male | 99,800 | 869 | 0.009 | -0.008 |
|  | Female | 21,256 | 189 | 0.009 | 0.008 |
| Lung | Male | 321,425 | 3,318 | 0.010 | 0.026 |
|  | Female | 158,646 | 1,550 | 0.010 | -0.026 |
| Pancreas | Male | 84,846 | 969 | 0.011 | 0.033 |
|  | Female | 74,920 | 801 | 0.011 | -0.033 |
| Liver | Male | 89,728 | 848 | 0.009 | -0.045 |
|  | Female | 35,483 | 370 | 0.010 | 0.045 |
| Colorectal | Male | 366,207 | 1,600 | 0.004 | -0.058 |
|  | Female | 252,792 | 1,241 | 0.005 | 0.058 |
| Biliary tract | Male | 43,994 | 205 | 0.005 | 0.104 |
|  | Female | 31,843 | 120 | 0.004 | -0.104 |

*Only cancer sites comparable to the National Hospital-based Cancer Registry for coverage rate were included. Sex-stratified national tabulations were publicly available for 2018–2021, whereas overall national counts used for Table 3 were available for 2018–2023; therefore, analytic windows differ across tables.

Supplemental Table S3 Availability of selected routine laboratory variables(2018–2023)

| **Test Item** | **Number of cases** | **Number of cases with available data** | **(%)** |
| --- | --- | --- | --- |
| CRE | 22847 | 18689 | 81.8 |
| AST/GOT | 22847 | 18580 | 81.3 |
| ALT/GOT | 22847 | 18624 | 81.5 |
| WBC | 22847 | 18670 | 81.7 |

This table is based on a subset of the J-CONNECT population described in Table 3.

The same cancer-site definition as Table 3 was applied, but the analysis was restricted to 8 of the 12 participating institutions included in the analytic dataset (N = 22,847).

Availability was defined as having at least one recorded result within 30 days of the initial diagnosis date.

These capture rates represent a feasibility-oriented description of laboratory data capture within this institutional subset and should not be interpreted as completeness estimates for the entire J-CONNECT population.
